# Effects of 12 Weeks Resistance Training on Serum Irisin in Older Male Adults

**DOI:** 10.3389/fphys.2017.00171

**Published:** 2017-03-22

**Authors:** Jiexiu Zhao, Zhongjun Su, Chaoyi Qu, Yanan Dong

**Affiliations:** ^1^Exercise Biological Center, China Institute of Sport ScienceBeijing, China; ^2^College of Physical Education and Health, Linyi UniversityLinyi, China; ^3^Department of Human Sports Science, Shanghai Sport UniversityShanghai, China; ^4^Department of Physical Education, Qufu Normal UniversityQufu, China

**Keywords:** irisin, body fat, resistance training, skin fold, dual-energy x-ray absorptiometry (DXA)

## Abstract

**Background:** To assess the effects of resistance training on circulating irisin concentration in older male adults, and to investigate the association between resistance training induced alteration of irisin and body fat.

**Methods:** Seventeen older adults (mean age is 62.1 years old) were randomized into old control group (male, *n* = 7), and old training group (male, *n* = 10). The control group has no any exercise intervention. The resistance training group underwent leg muscle strength and core strength training program two times/wk, 55 min/class for 12 weeks. Before and after the intervention, we evaluated serum irisin level and body composition.

**Results:** Serum irisin level was significantly increased in the resistance training group after the 12 weeks intervention period (*P* < 0.01), but not in the control group. In the resistance training group, the reduction in whole-body fat percent was negatively correlated with the increase in serum irisin level (*r* = −0.705, *P* < 0.05).

**Conclusion:** After the 12 weeks intervention, circulating irisin levels were significantly elevated in the older adults. In summary, serum irisin may be involved in the regulation of body fat in older male adults.

## Introduction

Obesity is recognized as a worldwide health concern (Reece et al., 2009; Wang et al., 2011). Fat accumulation induces obesity and increases the risk of type 2 diabetes, hypertension, and cardiovascular diseases (Abraham et al., 2015; Miyamoto-Mikami et al., 2015; Jiang et al., 2016). Regular physical exercise reduces or prevents fat accumulation throughout the body, although the detailed mechanism of which remains unclear (Slawta et al., 2002; Carroll and Dudfield, 2004; Zhao et al., 2011).

Irisin could be therapeutic for human metabolic diseases that are improved with exercise intervention (Bostrom et al., 2012). Irisin maybe is a human exercise gene (Timmons et al., 2012). It was demonstrated that peroxisome proliferator-activated receptor gamma coactivator 1-alpha (PGC1-a) expression might stimulate an increase in expression of FNDC5 expression in muscle (Bostrom et al., 2012). Irisin is a cleaved and secreted fragment of FNDC5 in circulating blood (Bostrom et al., 2012). Recent study demonstrated that irisin could up-regulate uncoupling protein 1 (UCP1) gene expression via in white adipose with exercise (Bostrom et al., 2012). Circulating irisin levels decrease with age (Huh et al., 2012; Tanisawa et al., 2014). Endurance training increases circulating irisin levels in middle-aged and older adults (Miyamoto-Mikami et al., 2015). It is unclear whether resistance exercise increases circulating irisin levels or not in older adults.

This study aimed to assess the effects of resistance training on circulating irisin in older male adults. We hypothesized that resistance training might increase serum irisin levels and reduce body fat in older male adults. To test our hypothesis, we investigated serum irisin levels and body fat percent in control and resistance training groups before and after intervention trial. In addition, we assessed the associations between circulating irisin levels and whole body fat in older male adults.

## Materials and methods

Seventeen older adults (mean age about 62 years old) were randomized into control group (male, *n* = 7), and resistance training group (male, *n* = 10). The subjects did not take any anti-hyperlipidemic, anti-hypertensive, anti-hyperglycemic medication. The control group has no any exercise intervention. The resistance training group underwent special resistance protocol. The present study was approved by the Research Ethics Committee of the China Institute of Sport Science (No. 20160039) and conducted in accordance with the Declaration of Helsinki.

### Study design

Before and after the 12-week resistance training period, body composition and serum irisin concentrations were measured. The venous blood samples (5 ml) were collected into pre-cooled vacutainer tubes at the beginning and end of the 12 weeks intervention period. Room temperature was maintained at 22°C throughout the experiment.

### Resistance training intervention

After baseline testing, resistance training groups participated in resistance-training program consisting of leg muscle strength and core strength training, the sequence is firstly leg muscle and then core strength, total duration 55 min/session (Sato and Mokha, 2009; Karavirta et al., 2011). The resistance training group underwent the training program including two times/wk for 12 weeks. The subjects were encouraged to maintain their usual levels of food intake during the whole experimental period (Miyamoto-Mikami et al., 2015).

### Body composition

Weight and height measurements were taken on the first test session (Jianmin II, Beijing Xin Dong Hua Teng, Beijing, China; Tian et al., 2015). Percent body fat (fat percent) was evaluated by dual-energy x-ray absorptiometry (DXA, GE LUNAR DPX system, Madison, WI, USA).

### Circulating irisin concentration

Circulating irisin concentrations were quantified by using the enzyme-linked immunosorbent assay (ELISA) kits (BioVendor-Laboratomi Medicina, Karasek, Crech Republic), according to the protocol of the manufacturer. Optical density at 450 nm was measured using a microplate reader (Thermo Scientific Multiskan MK3). Circulating irisin concentrations before and after the 12 weeks intervention of control and resistance training groups were simultaneously assessed after the 12 weeks intervention period. Other study proved that the assay is highly sensitive to human irisin (Lee et al., 2014).

### Statistical analyses

The results were expressed as means ± SD. Unpaired Student's *t*-tests was used for difference between the control group and resistance training group. Paired Student's *t*-test was used for comparisons of each parameter before and after training intervention. Pearson correlation coefficient was used to determine the strength of association between the amount of change in serum irisin and body composition parameter. The statistical calculations were performed using SPSS software for Windows (version 16.0, SPSS Inc., Chicago, IL). *P* < 0.05 denotes statistical significance.

## Results

### Difference of baselines in the training and control groups

There was no statistically significant difference in any parameter between the control group and resistance training group before the intervention (*P* > 0.05, Table 1). In the training group, body weight and fat percent were significantly decreased after resistance training (*P* < 0.05, Table 1). There was not significant change in body weight and fat percent after 12 weeks experiment in the control group (*P* > 0.05, Table 1).

**Table 1:** **Subject characteristics in old control and training groups**.

	**Control (*n* = 7)**		**Resistance training (*n* = 10)**	
	**Pre**	**Post**	**Pre**	**Post**
Age (years)	61.9 ± 3.1		62.3 ± 3.5	
Heights (cm)	165.7 ± 6.5		170.8 ± 6.1	
Body weight (kg)	71.2 ± 8.4	72.4 ± 9.0	76.4 ± 5.3	72.0 ± 4.4^*^
Fat percent (%)	31.8 ± 4.6	32.3 ± 4.7	27.6 ± 4.4	23.3 ± 2.9^*^^†^
Serum irisin level (ng/ml)	337.1 ± 137.8	327.1 ± 146.9	287.0 ± 143.5	556.0 ± 126.6^**^^††^

* *P < 0.05*,

** *P < 0.01: Pre vs. Post*.

† *P < 0.05*,

†† *P < 0.01: Resistance training group vs. control group*.

### Comparison of serum irisin concentrations between training and control groups

At baseline, there was no significant difference in serum irisin concentrations between training and control groups (*P* > 0.05, Table 1). After exercise-training intervention, serum irisin levels were significantly increased in the resistance training group (*P* < 0.01, and Table 1). After 12 weeks of resistance training, circulating irisin protein level in the resistance training group was significantly increased compared to that in the control group (*P* < 0.01, and Table 1).

### Correlation between serum irisin concentrations and body composition parameters

**Figure 1:**
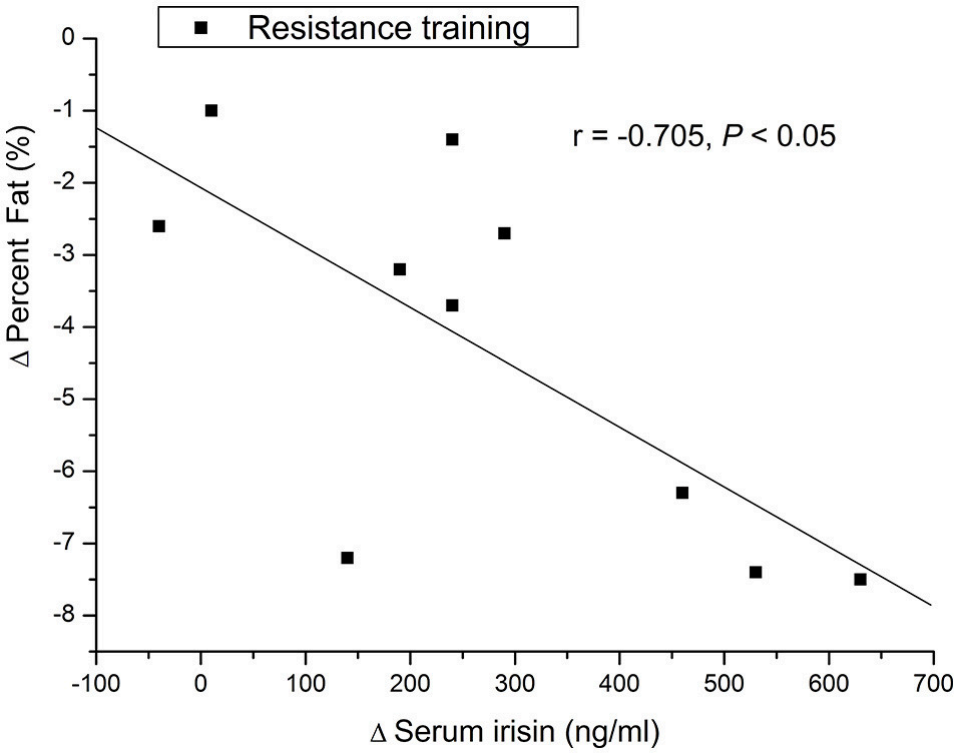
**Associations between change in serum irisin concentration and change in whole-body fat percent**.

**Figure 2:**
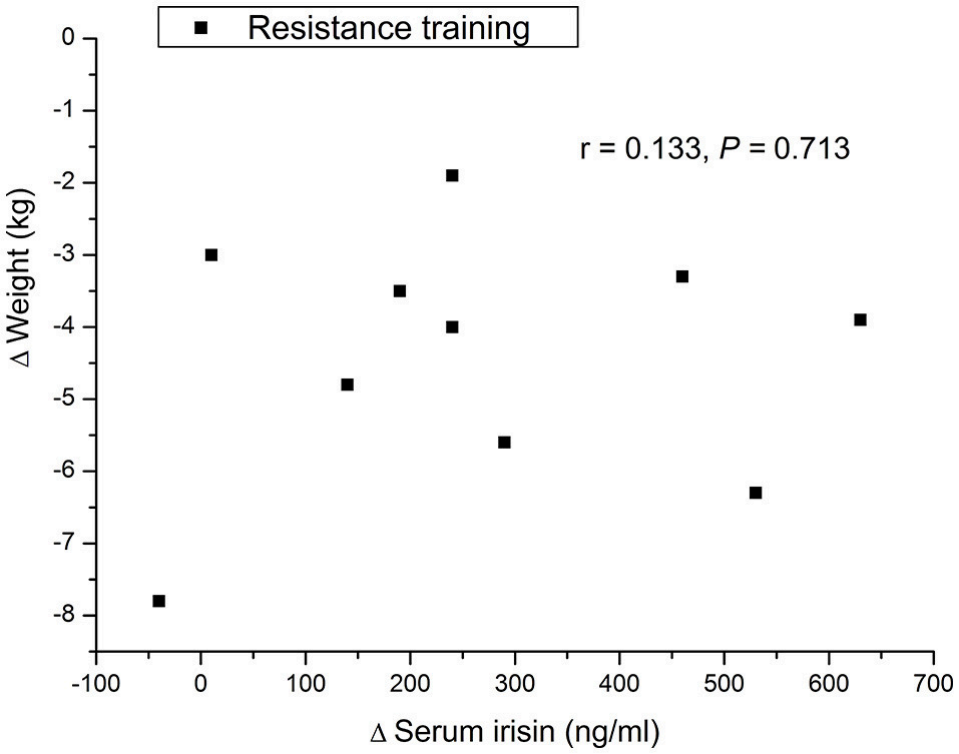
**Associations between change in serum irisin concentration and change in body weight**.

At baseline, there was no correlation between serum irisin concentration and body weight, height, or fat percent., There was a significant negative correlation between the increase in serum irisin concentration and the decrease in fat percent in the resistance training group (*r* = −0.705, *P* < 0.05, Figure 1). In addition, there was no significant association between the change in serum irisin and the change in body weight in the resistance training group or control group (Figure 2).

## Discussion

This study found two main aspects. First, serum irisin levels in older male adults were increased after the 12-week resistance-training, and this increase occurred accompanied with reductions of whole-body fat percent. Second, there was negative correlation between the change in circulating irisin levels before and after 12-week resistance training and the change in fat percent. It is demonstrated that the resistance training-induced change in serum irisin is accompanied with the change in body fat after 12-week resistance training in older male adults.

Chronic exercise training could have significant effects on circulating irisin levels in 12 randomized controlled trials (Qiu et al., 2015). Resistance training could counter decreases in muscle mass and strength in old adults (Kim et al., 2015). Meanwhile, previous studies suggested that exercise might induce irisin increase by contracting skeletal muscles (Bostrom et al., 2012; Kim et al., 2015). “FGF21-PGC-1α-Irisin axis” might be a potential mechanism accompanying with exercise (Sanchis-Gomar et al., 2012; Kong et al., 2014; Kim et al., 2015). In the present study, we found that there were lower fat percent and higher serum irisin levels in resistance training group than control group after 12 weeks experiment. We can draw a conclusion that the resistance training-induced change in circulating irisin is accompanied with the change in body fat after resistance training intervention in older adults. Previous study has showed that brown and beige fats can regulate fat metabolism by irisin in rat and human (Bostrom et al., 2012). These results together with other previous reports support that resistance exercise induced irisin might benefit the treatment of fat metabolism. This finding is in agreement with the conclusions of previous studies (Kim et al., 2015; Tsuchiya et al., 2015), which showed that resistant training might be an efficient intervention method to increase circulating irisin levels and resistance exercise resulted in significantly greater irisin responses compared with endurance exercise.

It is well known, that the increase of circulating irisin was found after endurance training intervention in human subjects (Bostrom et al., 2012; Miyamoto-Mikami et al., 2015; Qiu et al., 2015; Vosselman et al., 2015). However, it remains unclear whether resistance or strength training could affect circulating irisin. On the one hand, 8-week resistance training intervention did not affect serum irisin levels in healthy human subjects (Bang et al., 2014). On the other hand, 12-week resistance training intervention might be efficient method to increase the serum irisin level in mice and human subjects accompanied with improvement in muscle function (Kim et al., 2015). In this study, serum irisin was significantly increased after 12 weeks resistance training in older male adults. The results confirmed our hypothesis that the resistance training could restore reduction in circulating irisin level in older adults and high level of serum irisin might be associated with lower body fat in older adults.

This study has some limitations that should be considered. First, a possible limitation of the present study was the relatively small sample size and only male subjects. Therefore, the full effects of resistance training on serum irisin should be investigated in a large subject with both male and female older adults. Second, a dietary diary should be taken in order to exclude difference in caloric intake among the two groups which could affect the final results.

In summary, we demonstrated that serum irisin level was significantly increased in the resistance training group after the 12 weeks resistance training program in the older male adults. The increase in serum irisin levels was accompanied by the reduction of fat percent following 12 weeks resistance training intervention. Although the complete mechanisms should be tested in future studies, present study suggested that irisin may be involved in the regulation of body fat in older male adults.

## Author contributions

JZ, ZS, Study concept, design, data interpretation, data analysis and manuscript writing. CQ, YD, Study design, data analysis.

### Conflict of interest statement

The authors declare that the research was conducted in the absence of any commercial or financial relationships that could be construed as a potential conflict of interest.
